# Typology and Dynamics of Heavier Drinking Styles in Great Britain: 1978–2010

**DOI:** 10.1093/alcalc/agw105

**Published:** 2017-01-24

**Authors:** Robin C. Purshouse, Alan Brennan, Daniel Moyo, James Nicholls, Paul Norman

**Affiliations:** 1 Department of Automatic Control & Systems Engineering, University of Sheffield, Mappin Street, Sheffield, S1 3JD, UK; 2 School of Health & Related Research, University of Sheffield, 30 Regent Court, Regent Street, Sheffield, S1 4DA, UK; 3 Alcohol Research UK, 83 Victoria Street, London, SW1H 0HW, UK; 4 Faculty of Public Health Policy, London School of Hygiene and Tropical Medicine, London, WC1E 7HT, UK; 5 Department of Psychology, University of Sheffield, Cathedral Court, 1 Vicar Lane, Sheffield, S1 2LT, UK

## Abstract

**Aims:**

To identify a typology of heavier drinking styles in Great Britain and to identify socio-demographic trends in the typology over the period 1978–2010.

**Methods:**

We applied multiple correspondence analysis and agglomerative hierarchical clustering to beverage-specific quantity–frequency measures of alcohol consumption in the repeated cross-sectional General Lifestyle Survey of Great Britain, 1978–2010. The cluster analysis focuses on the 60,043 adult respondents over this period reporting average drinking levels above the UK Government guidelines. We projected sex, age, income, education, socio-economic status and tobacco consumption variables onto the clusters to inspect socio-demographic trends in heavier drinking.

**Results:**

We identified four stable clusters of heavier drinking: (a) high volume beer; (b) beer and spirit combination; (c) all beverage and (d) wine and spirit only. The socio-demographic characteristics of the clusters were distinct from both each other and the general population. However, all clusters had higher median incomes and higher smoking rates than the population. Increases in the prevalence of heavier drinking were driven by a 5-fold increase in the contribution of the female-dominated, wine and spirit only cluster.

**Conclusions:**

Recent changes in per capita alcohol consumption in Great Britain occurred within the context of a stable typology of heavier drinking styles and shifting socio-demographics. Identifying these trends is essential to better understand how drinking cultures develop over time and where potentially problematic drinking styles may emerge. Our findings suggest that careful attention to patterns and cultures of consumption is more important than relying on headline consumption data, for both understanding drinking behaviours and targeting interventions.

**Short Summary:**

This analysis of alcohol consumption survey data identifies four styles of heavier drinking in Great Britain, which remain unchanged over the period 1978–2010. The socio-demographic characteristics of the drinking styles are distinct from both each other and the general population, with increased participation of female and older drinkers over time.

## INTRODUCTION

The question of how and why population-level patterns of alcohol consumption change is of fundamental importance to alcohol research (Room, 1992; Room *et al*., 2009; Keyes *et al*., 2011; Bloomfield *et al*., 2016; Savic *et al*., 2016). In Great Britain, this debate has recently focused on understanding a long rise in per capita consumption from the early 1970s until the mid-2000s, which has been followed by a decade-long decline (British Beer and Pub Association, 2015). This decline has been partially driven by increased levels of abstention among young people and reduced levels of heavy episodic drinking (NatCen Social Research, 2015; Drummond *et al*., 2016). Analysing the dynamics of consumption and identifying underpinning factors is critical in understanding the nature of cultural change around alcohol. It is also important for the design and evaluation of interventions intended to reduce alcohol harm through modifying consumption. In particular, ongoing debates regarding the validity, and policy implications, of ‘whole-population’ approaches to consumption point to the need for refined tools by which we can analyse consumption patterns within aggregate population trends (Meier *et al*., 2009; Meier, 2010; Livingstone and Room, 2014; Norström and Svensson, 2014; Rehm, 2014; Rossow *et al*., 2014).

While some recent studies have offered conceptual models for making sense of changes within national drinking cultures (Room *et al*., 2009; Gordon *et al*., 2012; Savic *et al*., 2016), the main quantitative attempts to capture changing consumption dynamics have focused on age–period–cohort (APC) analyses and time-series methods. As surveyed by Keyes *et al*. (2011), APC methods have proved popular for describing trends in population-level consumption (e.g. mean consumption, abstention rates) in terms of age, period and birth cohort effects. However, these methods have been less successful at explaining the drivers of these trends, which has limited their usefulness for policy. For example, whilst Meng *et al*. (2014) identified a substantial period effect in females, the impact of, say, income as an explanatory factor is difficult to judge since it is used as a conventional control variable; therefore, its effect is averaged across all periods. A smaller number of studies have used time-series approaches to explain associations between current consumption and previous consumption (Brinkley, 1999; Alamani *et al*., 2014). Unfortunately, alcohol time-series studies have been hampered by the small number of time points available for analysis (Rehm and Gmel, 2001). Conventional methods struggle to identify factors that determine consumption change; they also tend to be restricted to evaluating relatively short-term changes, which are often contradicted by longer term ‘waves’ in consumption at the population level (Room, 1992; Room *et al*., 2016).

Whilst we might conceive of alcohol consumption as being composed of a rich tapestry of drinking occasions (see Mustonen *et al*., 2014; Ally *et al*., 2016), existing population-level studies of dynamics have tended to rely on very high-level metrics to describe patterns of consumption, such as per capita consumption (i.e. average grams of ethanol per person per day) or rates of abstinence or heavy drinking. Some studies seek to measure the role of short-term temporal variations (e.g. special occasions or weekends) in shaping consumption levels (Bellis *et al*., 2015), and a small number of studies have taken a step toward greater complexity by integrating beverage preferences into the analysis. Consideration of beverage preferences is important because the changes in per capita consumption seen over time tend to be underpinned by very different trends in the consumption of different types of beverage (e.g. beer, wine and spirit). Beverages can be affected by policy in different ways (e.g. beverage-specific tax regimes—see Ally *et al*. (2014)) and beverage preferences often differ between population subgroups and types of drinking occasion (Ally *et al*., 2016). Sulkunen (1989) conducted a descriptive analysis of French drinking, examining how the balance of three beverage classes (wine and cider combined, beer and spirit) had changed between 1965 and 1979 for different socio-economic and socio-geographic groups. The study found that reductions in wine consumption in lower socio-economic groups were preceded by similar changes in higher socio-economic groups. Kerr *et al*. (2004) conducted separate APC analyses for beer, wine and spirit for the US population between 1979 and 2000, identifying substantial beverage-specific birth cohort effects, including falling beer consumption in successive female cohorts and a peak in male spirit drinking in the early 1920s cohort (whose childhood coincided with the period of Prohibition).

In the present study, we aim to extend further the complexity of beverage-preference, population-level analyses to better understand the associations between alcohol consumption and social and economic change. To do this, we focus on one of the traditional consumption metrics used in the UK—the prevalence of weekly equivalent consumption at levels above the UK Government's safe drinking guidelines from 1987 to 2016—and identify further typologies within this based on beverage-specific drinking behaviour. We then identify the stability of both those typologies over time and the socio-demographic composition of the people who drink according to those styles.

## METHODS

### Data set

We use the General Lifestyle Survey (GLF), which is a repeated cross-sectional survey of UK households that collected data on alcohol consumption (using a beverage-specific quantity–frequency (QF) method) between 1978 and 2010 (Office for National Statistics. Social and Vital Statistics Division, 2012). Average weekly consumption responses are available for all even years, with the exception of 2004. High-level descriptive statistics are shown in Table 1. QF categorizations used in the analysis are shown in Table 2. GLF achieves approximately three-fifths coverage of population consumption against the gold standard measure of UK Government clearance data—the latter being archived by the British Beer and Pub Association (2015). GLF also includes data on a range of socio-demographic indicators—we use sex, age, household income and educational attainment variables to enable a comparison with Meng *et al*.’s APC analysis of the same data set. We do not include ethnicity due to problems with how this variable is recorded in early years of the GLF. We additionally include socio-economic status (SES) since, together with income, it is used in health equality analysis—specifically, the ‘alcohol harm paradox’ whereby deprived people are disproportionately affected by alcohol harms in relation to their levels of drinking (Beard *et al*., 2016). In terms of other behaviours, we include smoking since tobacco consumption and heavy alcohol consumption often occur in conjunction (Room, 2004) and may act as multiplicative risk factors (De Leon *et al*., 2007) for some types of cancer. Since some of the questions and codings in GLF have changed over time, preprocessing was required to produce a coherent data set—details of the procedures and the specific definitions of the socio-demographic variables are provided as Supplementary material.

**Table 1.** Alcohol consumption estimates from the GLF

Year	*N*	Population mean consumption (units^a^ per week)					Coverage^b^ (%	Prevalence of heavier drinking^c^	
		Beer	Wine	Fortified wine	Spirit	All		Increasing risk (%)	Higher risk (%)
1978	21,153	7.6	0.8	0.7	1.9	11.0	61	13.0	5.0
1980	21,159	7.2	0.9	0.7	1.8	10.6	58	12.7	4.6
1982^d^	18,576	6.5	1.0	0.6	1.7	9.8	58	12.6	3.8
1984	17,100	6.2	1.4	0.6	1.7	9.8	56	12.9	3.6
1986	17,850	6.5	1.6	0.5	1.8	10.5	59	14.0	4.0
1988	17,785	6.5	1.6	0.5	2.0	10.6	56	14.0	4.1
1990^e^	16,646	6.6	1.7	0.5	2.0	10.7	57	14.4	4.2
1992	17,301	6.4	1.8	0.4	1.9	10.4	58	14.9	3.6
1994	16,133	6.1	2.1	0.4	1.9	10.5	57	15.7	3.8
1996^f^	15,024	6.7	2.2	0.3	1.9	11.2	59	16.4	4.2
1998^g^	13,831	6.5	2.4	0.3	2.4	11.6	61	16.7	4.4
2000	13,500	6.4	2.7	0.3	2.8	12.2	61	17.8	4.8
2002^h^	14,143	5.8	2.9	0.2	2.9	11.8	55	17.6	4.8
2004^i^	0	5.6	2.8	0.2	2.5	11.2	50	16.2	4.2
2006^j^	15,918	5.9	5.4	0.2	2.2	13.8	65	20.0	6.9
2008^k^	14,007	5.2	5.1	0.1	2.1	12.6	60	18.4	5.9
2010	12,633	5.3	4.7	0.1	1.7	11.8	60	17.9	4.9

^a^1 UK unit = 8 g/10 ml ethanol.

^b^GLF adult per capita consumption estimate as a proportion of clearance data >14 years old per capita estimate [4].

^c^‘Increasing risk’ (>21–50 units per week for males; >14–35 units per week for females) and ‘higher risk’ (>50 units per week for males; >35 units per week for females) levels are defined according to the weekly equivalent UK Government guidelines on safe drinking levels 1987–2016.

^d^Question dropped on who was present during alcohol questions.

^e^‘Most days’ response split into ‘almost every day’ and ‘5–6 days per week’.

^f^Population weightings introduced.

^g^Changes to beverage questions: shandy dropped, ready-to-drinks introduced, beer split into low (<6% alcohol by volume) and high strength pints/cans/bottles.

^h^Longitudinal sample frame introduced.

^i^No QF questions.

^j^Increases in ethanol conversion rates: +0–1.5 units on beer servings, +1 unit wine serving.

^k^New serve sizes for wine (155/175/250/750 ml).

**Table 2.** QF categories used in the analysis for beverage types beer, wine, fortified wine and spirit

Quantity consumed per occasion (units)	Frequency of drinking occasion
Discrete scale based on serve sizes and conversion of natural volumes to ethanol. We group further to:	Re-interpreted qualitative categorization:
0	Never—‘not at all in last 12 months’
(0 1]	Rarely—‘once or twice a year/once every couple of months’
(1 2]	Monthly—‘once or twice a month’
(2 4]	Weekly—‘once or twice a week’
(4 8]	Some days—‘3 or 4 days a week’
>8	Most days—‘5 or 6 days a week’/‘almost every day’

To focus on the subpopulation drinking at levels above the UK guidelines during the period of the study, we analyse individuals reporting drinking at ‘increasing risk’ (>21–50 units per week for males; >14–35 units per week for females) and ‘higher risk’ (>50 units per week for males; >35 units per week for females) levels. One unit is equivalent to 10 ml or 8 g ethanol. Collectively, we refer to this subpopulation of individuals as ‘heavier drinkers’.

### Cluster identification

We use a two-step procedure to identify drinking clusters for each year of GLF data. Firstly, we reduce the dimensionality of the QF data and, secondly, we use formal clustering methods to identify groups in this lower dimensional space. The underpinning methodology follows previous work by Decullier *et al*. (2010).

We use the method of multiple correspondence analysis (MCA) to perform the dimensionality reduction (Le Roux and Rouanet, 2010). MCA is a categorical analogue to the more well-known method of principal component analysis for continuous data. The method aims to find a lower dimensional Euclidean representation of the data that maximally preserves the variance in the original data, where the individual response data for all QF questions have been converted to dummy variable format. The MCA procedure has been configured according to standard guidelines (Le Roux and Rouanet, 2010). Specifically, categories containing <5% of all observations are removed from the analysis, with the observations randomly reallocated to the remaining categories. The number of principal axes preserved in the lower dimensional space is chosen as the smallest number of axes that provides a cumulative modified variance rate of at least 0.8. Population weightings are not used in the analysis, but each sample is given a year weighting such that equal weight is given to each year. MCA has been conducted using the FactoMineR v1.24 implementation of MCA in R v3.0.2 (Husson *et al*., 2013).

Every respondent in the data can be projected on to the identified lower dimensional Euclidean representation to produce a so-called cloud of individuals (Lebaron, 2009). To identify clusters of drinking styles in the cloud, we apply Ward's minimum variance method (Everitt *et al*., 2001). This is an agglomerative hierarchical clustering technique that begins with each individual as his/her own cluster and then chooses two clusters to merge that provide the minimum increase in total within-cluster variance after merging. Ward's method assumes that the underlying clusters are both spherical and equally sized. Whilst visual inspection of the cloud suggests that these assumptions may not hold, alterative clustering techniques that relax the assumptions produce similar results (we investigated Gaussian mixture models, which allow for more general ellipsoidal clusters, and the DBSCAN algorithm, which allows for arbitrary cluster shapes (Everitt *et al*., 2001)). The clustering algorithm is applied to each year of GLF data in turn, with the thresholds used to define the cluster partitions chosen by inspection of the dendrogram of hierarchical clustering results. To follow clusters over time, we perform a matching based on the similarity of the cluster centroids between adjacent years. The clustering has been conducted in flashClust v1.01–2 in R v3.0.2 (Murtagh, 2013).

The population characteristics of the clusters are analysed by treating demographics as supplementary variables—such variables are simply treated as projections, and are not used to produce the clusters themselves. Post-processing of results has been performed in Matlab R2014a.

## RESULTS

### MCA results

MCA is applied to QF data for 60,043 heavier drinkers (45,195 increasing risk and 14,848 higher risk) over the period 1978–2010. The modified variance rates for the first three principal axes (*m*_1_, *m*_2_ and *m*_3_) of the MCA are 0.58, 0.18 and 0.09. These axes produce a cumulative rate of 0.85, which meets the threshold for selection of a reduced-dimension Euclidean space. Next we consider the contributions of the QF categories to each principal axis, and the direction of each of these contributions. In Table 3, the categories with greater than average contributions are shown. Axis *m*_1_ distinguishes between (a) bingeing beer consumption (>8 units per occasion) combined with abstention from other beverage types and (b) the consumption of wine (2–4 units most days) and fortified wine (up to 2 units possibly weekly). Axis *m*_2_ distinguishes between (c) frequent wine consumption combined with abstention from other beverages and (d) frequent, above daily guidelines beer consumption (4–8 units some days of the week). Axis *m*_3_ distinguishes between (e) weekly spirit consumption (at above daily guidelines or binge levels) combined with abstention from beer and (f) frequent wine consumption combined with occasional moderate beer drinking.

**Table 3.** Interpretation of principal axes: categorical variables are denoted by {quantity/frequency: beverage}

Axis	Negative coordinates	Positive coordinates
*m*_1_—Wine and fortified wine versus beer only Modified variance rate = 0.58	>8: Beer, never: wine, 0: wine, never: fortified, 0: fortified, never: spirit, 0: spirit	Rarely: beer, 0–1: beer, 1–2: beer, most days: wine, 2–4: wine, monthly: fortified, rarely: fortified, weekly: fortified, 0–1: fortified, 1–2: fortified
*m*_2_—Beer versus wine only Modified variance rate = 0.19	Some days: beer, 4–8: beer	Never: beer, 0: beer, most days: wine, never: spirit, 0: spirit
*m*_3_—Spirit versus wine Modified variance rate = 0.09	Never: beer, 0: beer, 0: fortified, weekly: spirit, 4–8: spirit, >8: spirit	Rarely: beer, monthly: beer, 0–1: beer, 1–2: beer, most days: wine, rarely: fortified, 0–1: fortified, never: spirit, 0: spirit

### Ward's minimum variance clustering results

**Fig. 1.**
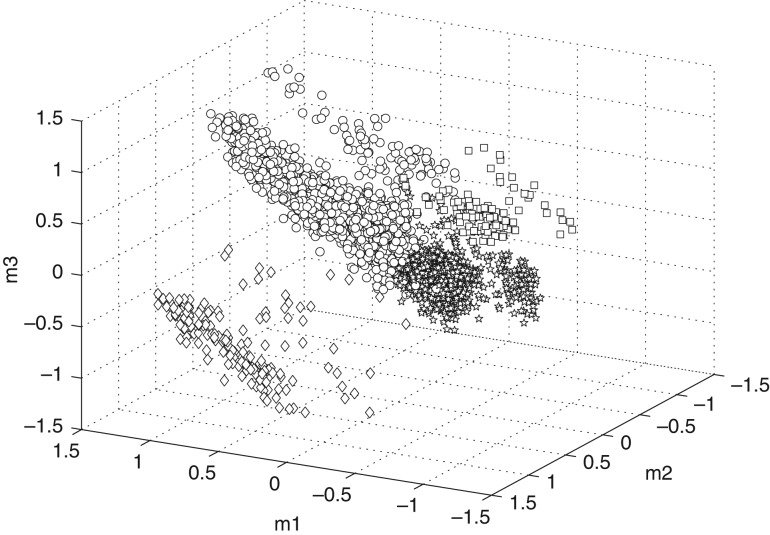
Clustered MCA projection for the cloud of individuals for GLF 1992. Square = high volume beer; star = beer and spirit combination; open circle = all beverage; diamond = wine and spirit only.

Ward's method suggests four potential clusters of drinking in each year of data. Illustrative findings for 1992 are shown in Figure 1. The two clusters representing the majority of heavier drinkers have heterogeneous beverage preferences. The largest cluster (45% of heavier drinkers, median weekly consumption of 32 units) has a centroid indicating a weak preference for beer drinking (at above-guidelines levels, potentially including binging) and weak preference for weekly spirit drinking (again at above-guidelines levels). We refer to this cluster as ‘beer and spirit combination’ drinking. The second cluster (35% of heavier drinkers, median weekly consumption of 27 units) has a centroid indicating preferences for multiple beverage classes (i.e. frequent consumption of beer, wine and spirit) and we refer to this cluster as ‘all beverage’ drinking.

Beer preferences form the major discriminator between the remaining, smaller clusters: the third cluster (14% of heavier drinkers, median weekly consumption of 33 units) has a centroid indicating a strong preference for binges on beer, with weaker preferences for wine drinking. The centroid of the fourth cluster (6% of heavier drinkers in 1992, median weekly consumption of 21 units) indicates a strong preference for frequent wine consumption combined with a moderate preference for weekly spirit drinking, both to the complete exclusion of beer. We consider these clusters as representing two drinking styles: ‘high volume beer’ drinking and ‘wine and spirit only’ drinking, respectively.

As suggested by the median weekly consumption levels, the majority of each cluster comprised increasing risk, as opposed to higher risk, drinkers. For example, in 1992, the relative prevalence of increasing risk consumption ranged from 77% for the ‘beer and spirit combination’ cluster to 87% for the ‘wine and spirit only’ cluster.

### Drinking dynamics

**Fig. 2.**
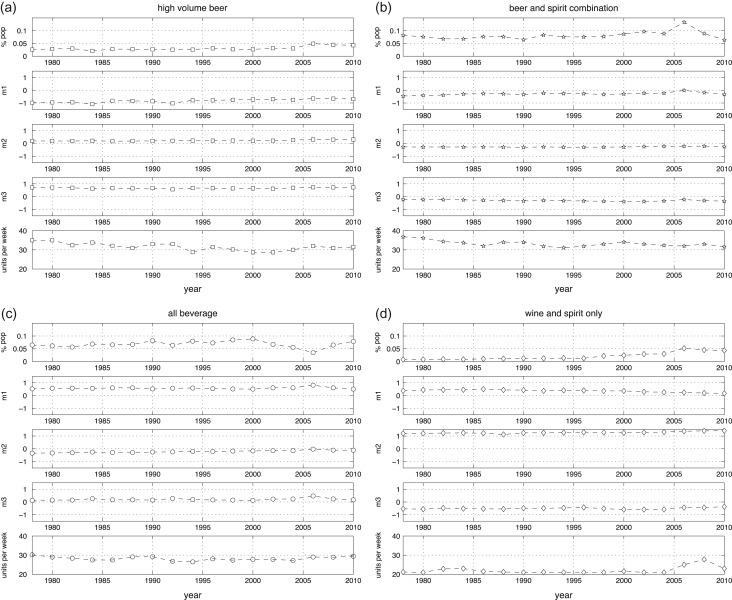
Cluster dynamics—drinking trajectories. Square = high volume beer; star = beer and spirit combination; open circle = all beverage; diamond = wine and spirit only.

Figure 2 shows, for each drinking cluster, (a) the size of the cluster, in terms of proportion of the overall British population involved; (b) the location of the cluster centroid according to the MCA principal axes and (c) the cluster median for weekly consumption.

As seen in Figure 2, for all four clusters, the location of the cluster centroid has remained stationary over time or followed a very slow trend, suggesting a strong degree of stability in the typology of heavier drinking between 1978 and 2010. Nevertheless, a substantial increase in prevalence is observed for ‘wine and spirit only’: rising from 0.8% of the population in 1978 to 4.3% in 2010. Consumption is observed to be stable in this cluster until 2006, when a revision to the wine serve size assumption used in GLF produces a step change in measured consumption (Goddard, 2007). This artefact is likely to actually correspond to a gentler trend, beginning in the mid-1990s.

### Drinker dynamics

**Fig. 3.**
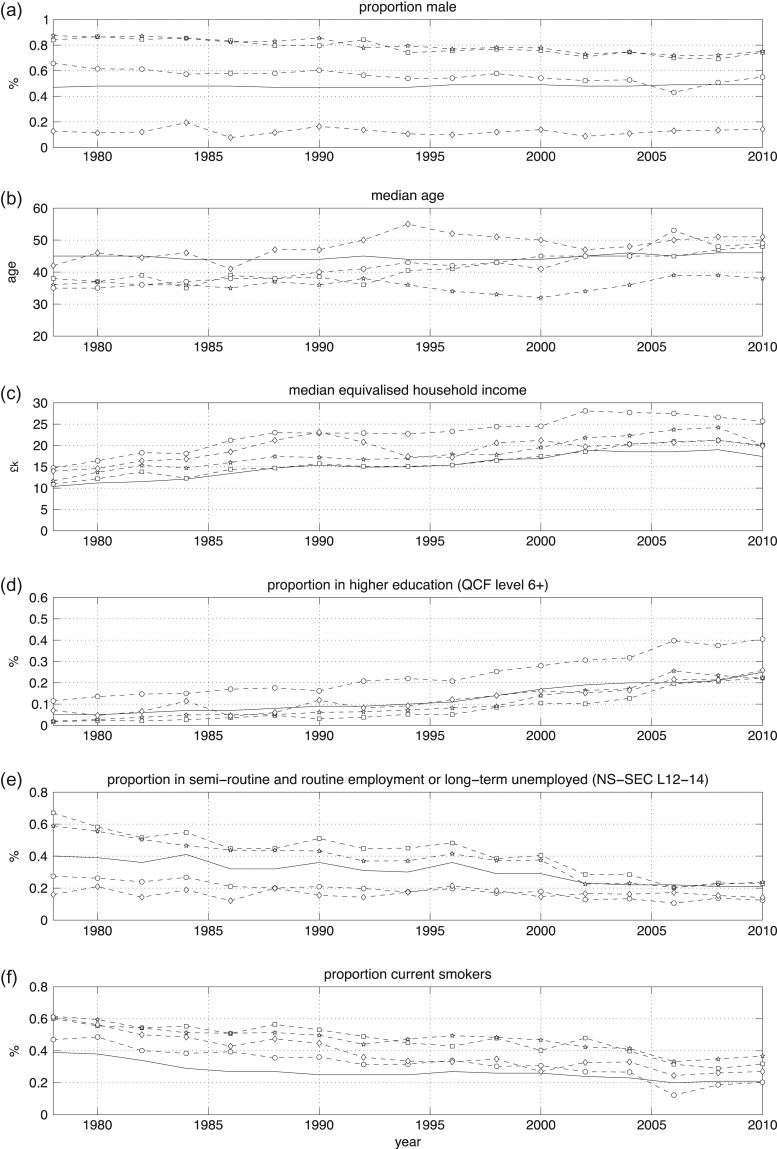
Cluster dynamics—drinker trajectories. Square = high volume beer; star = beer and spirit combination; open circle = all beverage; diamond = wine and spirit only.

Drinker dynamics for gender, age, income, education, SES and smoking, arising from analysis of the supplementary variables, are shown in Figure 3. Trajectories are shown for each cluster and also for the British population average.

#### Gender

From Figure 3a, it is clear that in 1978 one cluster (‘wine and spirit only’) is female dominated whilst the remaining clusters are male dominated. Whilst the three clusters with high male prevalence have become increasingly de-gendered over time (such that the ‘all beverage’ cluster has reached gender parity by the late 2000s), the ‘wine and spirit only’ cluster has retained a high female prevalence.

#### Age

As shown in Figure 3b, all four clusters have a median age below the population average in 1978, with ‘all beverage’ being the youngest cluster at a median 35 years of age. Three of the clusters have aged over time, with only ‘beer and spirit combination’ remaining below the population average age. The clusters have also grown in relation to the overall population over time, suggesting that additional older drinkers have been recruited into heavier drinking styles. The ‘wine and spirit only’ cluster exhibits rapid ageing during the first half of the 1990s—to a peak of 55 years of age in 1994—followed by a more gradual decaying trend back toward the population median over the late 1990s and early 2000s. Since the size of the cluster grows rapidly over this period of time, whilst the gender ratio remains unchanged, this is indicative of recruitment of first older, and then younger women into this drinking style.

#### Income

As shown in Figure 3c, all four heavier drinking clusters have median equivalised household incomes that are higher than or equal to the population average over the entire analysis period, with ‘high volume beer’ closest to the population average at £10,900 per annum in 1978 (in 2010 prices). Over the analysis period, all four clusters follow the population trend of increased real-term incomes. The 2008 recession is indicated by a reduction in incomes across all groups, and is particularly reflected in the steep fall for ‘beer and spirit combination’—the youngest cluster during this period.

#### Education

Results showing the proportion of individuals in each cluster who received a higher education (HE) are shown in Figure 3d. All four clusters follow the overall population trend of increasing HE participation over the analysis period. The ‘all beverage’ cluster has consistently higher participation levels in HE than the population average, whilst the ‘wine and spirit only’ cluster tracks the population average. Participation in the other two clusters is lower than the population average for much of the analysis period, but converges on the average by 2006, reflecting the expansion in HE provision in the UK since 1992.

#### Socio-economic status

Figure 3e shows the proportion of individuals in each cluster over time who are in semi-routine or routine labour, or who are long-term unemployed. Using this metric as a proxy for SES, we observe that, in 1978, two clusters have a higher proportion of individuals with a lower SES (67% for ‘high volume beer’ and 59% for ‘beer and spirit combination’) compared to the population average of 40%; meanwhile the other two clusters have a lower proportion (27% for ‘all beverage’ and 16% for ‘wine and spirit only’). Over the last 30 years, the composition of all four clusters reflects the observed general decrease in manual work, with both the ‘high volume beer’ and ‘beer and spirit combination’ clusters converging on the population average by the late 2000s.

#### Smoking

From Figure 3f, it is clear that all four heavier drinking clusters have higher than average rates of smoking than the population average (39%) in 1978. Three clusters have high prevalence rates (around 60%), whilst one cluster—‘all beverage’—has a lower rate of 47%. All four clusters follow the steady reduction in the prevalence of smoking seen in the general population over the last 30 years. Whilst the three higher prevalence groups remain above the population average, the ‘all beverage’ cluster appears to have converged on average smoking rates by 2000.

## DISCUSSION

### Comparison to existing knowledge on heavier drinking in Great Britain

The increasing prevalence of female heavier drinking seen in this study reinforces the conclusions of existing studies of population drinking dynamics—all of which have used GLF data. Kemm's (2003) birth cohort analysis of GLF 1978–1998 was the first to identify increasing prevalence of heavier drinking among females—specifically in birth cohorts from the 1930s and 1940s, with early indications of similar trends in 1970s cohorts. Smith and Foxcroft's (2009) descriptive analysis of GLF 1988–2006 provided more detail on the late 1970s and early 1980s cohorts—identifying a peak in the prevalence of female drinking in the 16–24 age group in the early 2000s. Most recently, Meng *et al*.’s (2014) APC analysis of GLF 1984–2009, whilst not focused on heavier drinking, also identified a consistently increasing period trend in average consumption by women, accompanied by a strong birth cohort effect showing younger women drinking more than older women—but with a decline, and potential reversal, of this trend among women born after 1985.

Our analysis adds more detail to this picture of female drinking dynamics. The increasing prevalence of heavier drinking in the 1930–1940s birth cohorts was strongly attracted to the existing female-dominant drinking style of ‘wine and spirit only’. This is evident from the ageing of this cluster during the early to mid-1990s when, according Meng *et al*. (2014), drinking by a woman born in the late 1930s would reach a second peak, subsequent to an initial peak in early adulthood. However, the second rise in heavier drinking—among women in late 1970s and early 1980s birth cohorts—appears to have not been confined to the ‘wine and spirit only’ style, but also to the ‘beer and spirit combination’ style, as evident from the decrease in median age observed for these clusters, in combination with the trend in de-gendering of the latter cluster. This provides some empirical evidence for the presence of the so-called ladette culture in Great Britain during the late 1990s and early 2000s (Day *et al*., 2004), but also indicates that other young women of this period opted for a style of heavier drinking that was similar to previous generations (i.e. not drinking beer). Interestingly, the quite substantive changes in female consumption did not give rise to any ‘new’ drinking styles—rather they were embedded within, or subtly extended, the existing heavier drinking typology. This finding supports Room *et al*.’s (2016) suggestion than consumption levels are more likely to change over time ‘within’ a given culture, rather than there being dramatic shifts in drinking styles. The continuing reductions in consumption seen since 2010 (e.g. reduced participation in alcohol use among young people, reductions in heavy episodic drinking, and reductions in increasing risk consumption in men) are likely to change the drinker trajectories of each heavier drinking style (e.g. the clusters are likely to age), but it remains to be seen if they will be accompanied by any tangible changes to the typology itself.

### Historical perspectives

This analysis brings us new perspectives on developments in British drinking cultures since the 1980s. Over this period, both the legislative framework and market structures around alcohol have changed dramatically (Nicholls, 2009; Spicer *et al*., 2013). Most notably, there has been a major shift in where people drink: the proportion of beer sold in pubs and bars, for instance, declined from 88% in 1980 to 50% in 2014 (British Beer and Pub Association, 2015). This shift in drinking location has been accompanied by a change in the beverages that people consume, driven predominantly by a dramatic increase in the wine market. Wine consumption rose from 8.1 l per capita in 1980 to a peak of 23 l per capita in 2007 (British Beer and Pub Association, 2015): an increase of 184%, as compared to a fall of 26% in the volume of beer consumed per adult over the same period. This trend reflects changes in the international wine market, particularly the expansion of ‘new world’ wine imports (Hurley, 2005), but also the marked increase in consumption among women seen in this and other analyses (Kemm, 2003; Smith and Foxcroft, 2009; Meng *et al*., 2014). Another notable trend is the extent to which wine has been, as it were, democratized. Until the 1990s, the cluster most strongly associated with wine (‘wine and spirit only’) was one of the two affluent groups of drinkers identified in this analysis. Until 1990, this cluster had a median household income significantly above the population average; however, by the mid-2000s, the average income of this group was close to the population median. This dynamic, driven by increased female consumption across varying birth cohorts, reflects a lowering of prices in the wine market and an expansion of availability in large supermarkets, small convenience stores and, more recently, online retailers. Wine is no longer a product sold primarily through specialist outlets to an affluent (though often high-consuming) market: it is now widely available, affordable and popular across a range of social groups. Important developments also occurred in the beer market over this period, particularly the promotion of continental lager brands with greater alcohol content than their domestic competitors and, latterly, the resurgence of the market for craft beers.

The long increase in per capita consumption between 1980 and the mid-2000s (an increase which began in the early 1970s) was driven, primarily, by an expanding wine market but also by higher strength beers and the development of new products, including—but only to a limited extent—ready-to-drink (RTDs) mixers, also known as ‘alcopops’. Until now, however, our understanding of the extent to which these trends can be attributed to high-consuming subgroups has been limited. This has meant that debates on the relationship between population trends and harmful consumption have, outside of the specialist research community at least, often relied on somewhat crude assumptions regarding overall proportions of moderate, hazardous and harmful drinking in the population. At the same time, much media reporting have reinforced the incorrect assumption that consumption increases are isolated primarily among young people consuming RTDs, lager and spirits (Nicholls, 2011; Atkinson and Sumnall, 2011). By contrast, our analysis points to a more complex picture in which choice of drink, pattern of consumption and socio-economic contexts play a critical role as determinants of potential harm. This does not, by itself, call into question the models that suggest a structural relationship between overall population consumption levels and harmful consumption (Skog, 1985). It does, however, contribute to our understanding of the patchwork of drinking behaviours among those groups where harms tend to be concentrated.

### Limitations

MCA and clustering are exploratory data analysis methods that lack the formal hypothesis testing instruments associated with more conventional analysis methods such as APC and time series. Therefore, it is important to avoid making too strong an interpretation of our findings. However, we stress that the absence of goodness-of-fit metrics and significance tests needs to be balanced against the advantages of MCA and clustering for identifying nonlinear relationships in the data—a key concern for alcohol research. We have been able to identify dichotomies and dynamics (e.g. the SES characteristics in Figure 3e) that are at risk of being obscured or misinterpreted using conventional methods.

Our analysis has focused on the central tendencies associated with the clusters (i.e. centroids, median properties and simple binary demographic splits). However, it is possible that higher order moments and richer categorical demographic indicators might modify some of the conclusions. Further limitations relate to issues with the GLF itself. Since the survey is not longitudinal, we cannot track the relative stability of individuals within clusters over time. In the long-run data, the QF questions are separated by beverage, so drinking occasions that comprise beverage combinations cannot be identified, and the diversity of drinking occasions that can be identified is also limited (Boniface *et al*., 2014; Bellis *et al*., 2015). The survey was slow in accounting for larger serve sizes and increasing beverage strengths during the late 1990s and early 2000s.

It is important to recognize that GLF does not capture all of the population subgroups that might include heavier drinkers; people who are homeless or are resident in institutions, care homes or prisons are not sampled, whilst students and dependent drinkers are under-sampled (Meier *et al*., 2013). These groups may exhibit different styles of drinking to those identified from GLF, or may exhibit similar styles but possess different socio-demographic characteristics. The four clusters of heavier drinking identified in the paper all have median incomes greater than the population average. However, this finding could be tempered by inclusion of homeless heavier drinkers (analysis of the 1994 Psychiatric Morbidity Survey (Office of Population Censuses and Surveys. Social Survey Division, 1997) suggests that 12.1% of homeless individuals drink at higher risk levels, compared to the GLF estimate for private households of 3.8%). Improved representation of dependent drinkers may also temper this finding; whilst the 2007 Psychiatric Morbidity Survey found no clear relationship between income and dependency (Fuller *et al*., 2009), this too was a household survey so will suffer from similar issues to the GLF.

### Toward a causal account of consumption change and stasis

Drinking typologies tend to be conceived in the literature as classes of developmental trajectories for individuals, typically identified from cohort studies; see Sher *et al*. (2011) for an interesting review. Recent research has drawn attention to the importance of considering drinking occasions, and the styles associated with those occasions, in analysing cultural trends (Ally *et al*., 2016). There have also been calls for greater attention to be drawn to the divergent patterns of consumption ‘within’ populations (Savic *et al*., 2016; Pennay *et al*., 2015; Norström and Svensson, 2014; Meier, 2010). Our analysis provides a complementary perspective, by considering each identified drinking style as a ‘case’ in its own right, with its own trajectory over time (Byrne, 1998). Descriptor variables for each case (e.g. cluster centroid or size) can then be used in longitudinal analyses—e.g. they can be used as dependent variables in an, otherwise conventional, time-series analysis. In this way, the approach can highlight how broad styles of drinking, and the characteristics of the population subgroups that drink according to those styles, develop and endure over time. By mapping these dynamics against policy and other socio-economic changes, future research may be able to further refine our existing models for the interactions between context, levels and styles of consumption within populations.

## Supplementary Material

Supplementary Data
