# Supplementary material for: Typology and Dynamics of Heavier Drinking Styles in Great Britain: 1978–2010
Source: Alcohol Alcohol. 2017 Jan 24;52(3):372–81. doi: 10.1093/alcalc/agw105 (PMC5397881; doi:10.1093/alcalc/agw105)
Supplement: Supplementary Data [file 20161125macroanarevisedversion.docx]

**S. Supplementary Information**

**S.1 Data pre-processing**

Some of the questions and codings in GLF have changed over time, including the types of beverage for the QF questions and minor changes to the frequency categories themselves. In the analysis we work with four primary beverage types: beer (including shandy, normal strength beer and strong beer), wine, fortified wine and spirit (including ready-to-drink beverages, also known as flavoured-alcoholic-beverages or alcopops). In instances where QF data exists for an individual for multiple beverages within one of the primary types (e.g. normal strength beer and strong beer simultaneously), we use the QF categories that are associated with the most ethanol consumption.

In addition to the beverage information, we include a number of demographic indicators: sex, age, income (using a gross equivalised household definition, deflated to 2010 prices), highest level of educational qualification achieved (using QCF^[[1]](#footnote-1)^ definitions), socio-economic status (using NS-SEC^[[2]](#footnote-2)^ definitions), and current smoking status. For education and socio-economic status we use the latest codings available (those for GLF 2010) and necessarily map earlier definitions to these latest versions; this requires a degree of qualitative interpretation. Respondents who answer inconsistently to drinking questions are removed from the analysis. QF questions were not asked in GLF 2004 and so synthetic data is created for this year by combining data from adjacent years where QF data is available (single-weighted 2002 combined with double-weighted 2005). All pre-processing has been performed in Stata v12.

1. http://www.qaa.ac.uk/Publications/InformationAndGuidance/Documents/Quals_cross_boundaries.pdf [↑](#footnote-ref-1)
2. http://www.ons.gov.uk/ons/guide-method/classifications/current-standard-classifications/soc2010/soc2010-volume-3-ns-sec--rebased-on-soc2010--user-manual/index.html [↑](#footnote-ref-2)
